# Pilot evaluation of a socio-emotional learning program on executive functions in elementary school students: a cluster-randomized controlled trial

**DOI:** 10.3389/fpsyg.2025.1554001

**Published:** 2025-07-28

**Authors:** Rita Antunes, Tânia Carneiro, Luciana Joaquim, Margarida Matos, Joana Alexandre, Marisa G. Filipe

**Affiliations:** ^1^Center for Research in Psychology for Development, Lusíada University of Lisbon, Lisbon, Portugal; ^2^Neurodevelopment Unit at CUF Descobertas Hospital, Lisbon, Portugal; ^3^Center of Linguistics, School of Arts and Humanities, University of Lisbon, Lisbon, Portugal; ^4^Colégio São José de Bairros, Lousada, Portugal; ^5^Agrupamento de Escolas Irmãos Passos, Matosinhos, Portugal; ^6^ISCTE, University Institute of Lisbon Centre for Psychological Research and Social Intervention (CIS_ISCTE/CVTT), Lisbon, Portugal

**Keywords:** social and emotional learning program, elementary school, socio-emotional competencies, executive functions, pilot study

## Abstract

Social and Emotional Learning (SEL) programs aim to enhance children’s socio-emotional competencies. While research has highlighted the positive socio-emotional outcomes of SEL programs, their transfer effects on executive functions remain unclear. This pilot cluster-randomized controlled trial explored the impact of a SEL program – *The Intergalactic World* – on executive functions in a cohort of first to fourth-graders. Participants were randomly assigned to either an intervention group (*n* = 60; 26 girls; *M*_age_ = 94.95 months, *SD* = 15.53), which participated in the SEL program, or to a waiting list control group (*n* = 36; 19 girls; M_age_ = 111.62 months, *SD* = 6.80). The SEL program comprised eight sessions conducted in a school setting to enhance socio-emotional development through breathing exercises, relaxation techniques, guided imagery, and cognitive-behavioral strategies. Executive functions were assessed for both groups using a standardized questionnaire, with evaluation conducted before and after the program implementation by teachers and caregivers. Pre- and post-intervention assessments revealed no significant changes in executive function scores based on teacher ratings across groups. In contrast, caregiver ratings of executive functions showed a significant interaction effect between time and group, with the intervention group exhibiting a positive change over time compared to the control group. Results yield valuable insights into the potential benefits of SEL interventions concerning elementary school students’ executive functions.

## 1 Introduction

Social and emotional competencies play a critical role in promoting positive life outcomes, encouraging prosocial behaviors, and reducing behavioral issues (Kankaraš and Suarez-Alvarez, 2019; Singh and Duraiappah, 2020). The Collaborative for Academic, Social, and Emotional Learning (2012) defines Social–Emotional Learning (SEL) as “the processes through which children and adults acquire and effectively apply the knowledge, attitudes, and skills necessary to understand and manage emotions, set and achieve positive goals, feel and show empathy for others, establish and maintain positive relationships, and make responsible decisions” (p. 9). These competencies are crucial not only for academic success but also for overall well-being (Hassani, 2024).

Research has extensively analyzed the development of social and emotional skills within school contexts, showing that these particular skills can be intentionally taught, modeled, and practiced through structured interventions, positively affecting student behavior and academic performance (Cipriano et al., 2023; Weissberg, 2019). The effectiveness of SEL interventions can vary significantly depending on several contextual and implementation factors. These include the age of the students, with evidence suggesting that effects are generally stronger in younger children (Taylor et al., 2017), as well as the duration of the intervention, its cultural appropriateness, and the involvement and training of teachers (Fernández-Martín et al., 2021; Mahoney et al., 2021).

Systemic approaches to SEL stand out for their sustainability and wide-ranging impact. According to Mahoney et al. (2021), integrating SEL into educational policies and teacher training ensures lasting benefits, such as improved academic performance and emotional regulation in diverse student populations. These systemic efforts seek to position SEL programs as a core element of education, preparing students to navigate the complexities of academic and social challenges effectively (Zhang and Ma, 2023).

Although SEL programs are widely endorsed for promoting student well-being, evidence regarding their effectiveness remains mixed. Meta-analyses have shown that students who participated in SEL programs demonstrated significant improvements in emotional well-being, reduced stress levels, and enhanced academic performance compared to their non-participating peers (Durlak et al., 2022; Taylor et al., 2017). However, some studies frequently encounter challenges related to implementation fidelity and the consideration of contextual factors such as culture and school climate. Klingbeil et al. (2017) highlight the scarcity of studies assessing implementation fidelity in mindfulness-based interventions (i.e., a subtype of SEL program according to some authors Klingbeil et al., 2017; Lawlor, 2016), which undermines the validity of findings and complicates efforts to determine their influence on program effectiveness. This methodological limitation is also observed in executive functions (EFs) research, which are increasingly recognized as crucial for socioemotional development. Similar to SEL programs, studies on EFs often overlook contextual variables that may significantly influence outcomes. For instance, Lewis et al. (2009) emphasize that measures of EFs and social cognition do not always translate appropriately across cultures. This absence of fidelity data, combined with the impact of culture and contextual factors, limits conclusions regarding the extent to which treatment integrity may moderate effectiveness.

The cultural adaptation of SEL programs has demonstrated their applicability across diverse contexts. Interventions tailored to the specific needs and values of local communities have been shown to be more effective, emphasizing the importance of aligning SEL approaches with the cultural and social characteristics of the target groups (OECD, 2024). These adaptations promote the development of core skills such as empathy, emotional regulation, and problem-solving, which are essential for success in different contexts (Cipriano et al., 2023; Domitrovich et al., 2017). In the Portuguese context, empirical research on SEL is gradually emerging (Antunes et al., 2022; Antunes et al., 2023; Cristóvão et al., 2017). However, the limited number of studies and the variability in reported outcomes suggest that broad generalizations should be made with caution. Recent findings indicate that the effectiveness of SEL interventions in Portugal may be shaped by several contextual factors, including educator training and regional disparities. Moreira et al. (2010) highlighted the need to consider such variables in future research, given their potential role as mediators of program outcomes. Moreover, cultural norms regarding emotional expression, disciplinary practices, and teacher-student relationships are key moderators of program impact. These considerations underscore the importance of culturally responsive implementation strategies that account for the sociocultural specificities of educational settings to ensure the relevance and effectiveness of SEL initiatives.

The COVID-19 pandemic has heightened the visibility and urgency of SEL programs, particularly in addressing increased socio-emotional difficulties, mental health disparities, and educational inequities among students (Office of the Surgeon General, 2021). In this context, SEL has emerged as a key strategy for promoting resilience and supporting student adjustment to the complex demands of the post-pandemic educational landscape (Grazzani et al., 2022; Raimundo et al., 2024).

In exploring the mechanisms that support socio-emotional development, EFs emerge as a critical domain (Riggs et al., 2006), which represent higher-order cognitive processes essential for self-regulation, goal-directed behavior, and adaptive functioning. Although EFs lack a universally agreed-upon definition due to its complex and multidimensional nature, it generally encompasses attentional control, cognitive flexibility, planning, goal setting, inhibition, and self-regulation (Anderson et al., 2002; Jurado and Rosselli, 2007; Miyake et al., 2000). These functions develop progressively throughout childhood and adolescence, supporting a child’s ability to manage thoughts, emotions, and behaviors (Anderson et al., 2002).

Riggs et al. (2006) emphasize that EFs, for instance impulse control and problem-solving, are closely linked to social–emotional competencies. Indeed, research has shown that deficits in EFs can contribute to internalizing problems and increased stress reactivity (Thompson et al., 2019), potentially impairing children’s ability to engage effectively with their environment and resulting in social difficulties (Clark et al., 2002). Specifically, children with lower EFs scores often exhibit poorer social–emotional skills and encounter more significant behavioral challenges than their peers with higher EFs scores (Dias et al., 2017; Riggs et al., 2004).

The relationship between SEL and EFs is dynamic and multifaceted. Neuroimaging and behavioral studies suggest that emotional regulation and EFs may be interrelated, as they rely on partially overlapping neural systems involved in cognitive control (Sudikoff et al., 2015). SEL interventions targeting emotional and attentional processes could also influence components of EFs (Zelazo and Lyons, 2012). However, the current evidence remains largely correlational, and direct causal links are not yet well established.

Some SEL programs, particularly those that include explicit components such as self-monitoring, planning, and reflective practices, have been shown to support the development of EFs (Diamond and Lee, 2011). This suggests the need to distinguish between the broader socio-emotional benefits of SEL (e.g., increased empathy, prosocial behavior, and school engagement) and its more specific cognitive outcomes, particularly in relation to executive functioning.

Within this context, mindfulness-based SEL interventions have received growing attention for their potential to enhance EFs. Emerging evidence indicates that when well-structured and developmentally appropriate, these interventions can foster improvements in core EFs, including attentional control and cognitive flexibility, especially among children with lower baseline EFs skills (Diamond and Lee, 2011). For instance, Flook et al. (2010) found positive effects of mindfulness-based practices on EFs outcomes in school-aged children. Similarly, after a mindfulness-based intervention, Ritter and Alvarez (2020) reported significant improvements in inhibition, working memory, and cognitive flexibility among elementary students. In addition, Crooks et al. (2020) evaluated the MindUP program, a mindfulness-based SEL intervention, and observed reductions in behavioral symptoms, internalizing and externalizing behavior problems, EFs deficits, and increases in adaptive skills. Thierry et al. (2016) also found that preschool children participating in MindUP showed gains in teacher-reported EFs-related behaviors, particularly in working memory, planning, and organizing.

### 1.1 Present study

Although previous research has consistently demonstrated the positive effects of SEL programs on socio-emotional skills, their potential to influence cognitive domains, such as EFs, remains understood. While mindfulness-based interventions have shown positive outcomes on EFs, their classification as a subset of SEL programs is debated. Mindfulness interventions emphasize enhancing self-awareness and attentional regulation “from the inside out,” focusing on internal experiences such as thoughts and emotions. In contrast, SEL interventions aim to foster emotional regulation and skill development “from the outside in”, targeting improved academic performance and reduced risk behaviors (Semple et al., 2017). This nuanced distinction raises the possibility that the mechanisms of influence on EFs may differ between these intervention types.

Adding to this complexity is the scarcity of research on SEL interventions’ impacts in culturally specific contexts. While the efficacy and effectiveness of SEL programs have been predominantly studied in Anglo-Saxon countries, limited research has been conducted in other regions (Fernández-Martín et al., 2021). Addressing this gap is crucial for developing evidence-based programs that account for the unique needs of diverse student populations. Tailored interventions offer an opportunity to understand the interplay between SEL and EFs in contexts that remain underexplored in the literature.

This pilot cluster randomized controlled trial explored the effects of a SEL intervention on first to fourth-grader students, focusing on the transfer effects on EFs in a Southern European country context – Portugal. Based on the reviewed literature, we proposed the following hypothesis: Participation in a SEL program will significantly improve children’s EFs, as assessed by caregiver and parents’ ratings, compared to a control group. This pilot study is also innovative in exploring the transfer effects of SEL on EFs because it emphasized the importance of ecologically valid assessment methods, such as ratings provided by caregiver and teacher ratings.

Effective EFs assessment is critical for understanding their influence on academic and socio-emotional outcomes. While performance-based assessments—conducted in structured settings—offer insights into EFs skills, they often fail to reflect how these skills are applied in real-life contexts. Thus, ecologically valid measures, such as questionnaires and behavioral ratings, have gained prominence for their ability to capture EFs functioning in daily environments from the perspectives of parents, teachers, or other observers (Silver, 2014; Toplak et al., 2013). These measures are especially relevant for children, as EFs-related behaviors and challenges can vary significantly between contexts like home and school. Questionnaires help identify EFs deficits that standardized testing might overlook, providing a more comprehensive understanding of EFs, especially in populations such as children with neurodevelopmental disorders, where EFs difficulties are often context-dependent (Soriano-Ferrer et al., 2014; Tan et al., 2018).

By addressing these gaps, this pilot study aimed to contribute to our understanding of how SEL interventions contribute to developing socio-emotional and EFs skills in a less-studied cultural context. These findings can inform the design of integrated educational programs that promote socio-emotional and cognitive development, holding the potential to inform the design of integrated educational programs that holistically support children’s socio-emotional and cognitive development.

## 2 Methods

### 2.1 Participants

A cluster randomized controlled trial design was employed, with convenience sampling based on the school’s accessibility and willingness to participate in the study. Ninety-six children (45 girls, 51 boys; *M*_age_ = 101.20, *SD* = 15.26) from one private school in the Porto Metropolitan Area, Portugal, participated in this study. Participants were 1st to 4th graders, distributed as follows: 1st grade – 23 students (one class), 2nd grade – 21 students (one class), 3rd grade – 19 students (one class), and 4th grade – 33 students (two classes). Class sizes ranged from 16 to 23 students, with an average of 19.2 students per class (*SD* = 2.86). According to school reports, the majority of children were typically developing native speakers of European Portuguese. However, one student in 2nd grade, one in 3rd grade, and three in 4th grade presented specific educational needs and required selective and additional measures at school.

Participants were recruited by the psychologists who delivered the intervention program in their host school. Classes were randomly assigned to either the intervention group or the waiting list control group. Randomization was performed using Microsoft Excel by generating random numbers, ensuring an unbiased allocation in a simple and transparent manner. The intervention group consisted of 60 students (26 girls; *M*_age_ = 94.95 months, *SD* = 15.53), who received the SEL program. The waiting list control group included 36 students (19 girls; *M*_age_ = 111.62 months, *SD* = 6.80). The academic performance of students in the two main subjects, namely Mathematics and Language, was comparable between the control and intervention groups, with minor differences in the percentage of students achieving the highest classification (“Excellent”) 55.56% vs. 47.46% in Mathematics and 54.24% vs. 47.22% in Language, respectively. The demographic characteristics of the sample are presented in Table 1.

**Table 1:** Demographic characteristics of students by group.

Variable	SEL	Control
Participants (*n*)	60	36
Age (months)		
*M*	94.95	111.62
*SD*	15.53	6.80
Gender		
Boys	34	17
Girls	26	19
Education level		
1st grade (*n*)	23	*-*
2nd grade (*n*)	21	*-*
3rd grade (*n*)	*-*	19
4th grade (*n*)	16	17

### 2.2 Socio-emotional learning program: *The Intergalactic World*

*The Intergalactic World* was developed to address the increasing need for SEL interventions for elementary and secondary school children in Portugal (Antunes et al., 2022, 2023). This program aligns with evidence supporting cognitive-behavioral interventions as effective in promoting social–emotional competencies, particularly self-control (Smith et al., 2019). This universal program consisted of eight sessions aimed at enhancing self-regulation, self-control, and attentional focus. Grounded in existing literature (e.g., Sanders, 2008; Webster-Stratton, 2016), the program combines psychoeducational methods with play-based therapeutic approaches to foster a supportive learning environment. The program included relaxation exercises along with cognitive-behavioral training (e.g., Black and Fernando, 2014; Ferraioli and Harris, 2013; Huguet et al., 2017; Raveepatarakul et al., 2014; Vickery and Dorjee, 2016).

Previous studies have demonstrated that this program benefits younger (8–9 years) and older (10–12 years) children, with reductions in psychopathological symptoms, such as anxiety, depression, and stress, as well as enhancements in socio-emotional skills from pretest to posttest (Antunes et al., 2022, 2023). Follow-up assessments also confirm the lasting benefits (Antunes et al., 2022, 2023). Details of the intervention program, including session-by-session content, are provided in Table 2.

**Table 2:** The intergalactic world: aims, activities, and key contents for each session.

Session number	SEL domains	Activities	Key contents
1	Self-knowledge Self-management Social consciousness Interpersonal relationship	Welcome Guidelines Introduce group leaders Relaxation activity ‘My Intergalactic Passport’ ‘My Superpowers’: Discovering my inner Superhero ‘My Superpowers’ (Super Adventure)	Relaxation dynamics using deep breathing and music Reflection on personal characteristics, behaviors, and feelings Discover and share personal potential
2	Self-management Social consciousness Interpersonal relationship	‘An Intergalactic Day’ ‘School in the Galaxy of Behavior’ ‘My Superpowers’ (Super Creativity)	Reflection on daily vs. ideal routines (school/home) Sharing of interests via drawing/free writing Mime exercise and reflection in small groups
3	Self-knowledge Self-management Social consciousness Responsible decision-making	‘The Theater of Intergalactic Emotions’ ‘The Intergalactic Mirror’ My Superpowers’ (Super Energy)	Reflection on emotions using images of 4 basic emotions (happiness, sadness, fear, anger) Group exercises: face-to-face ‘mirroring’ of movements, incorporating music, rhythm, and speed
4	Self-knowledge Self-management Social consciousness Interpersonal relationship Responsible decision-making	‘Intergalactic Relaxation’ ‘An Apple at the Intergalactic World’ ‘My Superpowers’ (Super Attention)	Relaxation through imagery and dramatization (e.g., astronaut movement) Emotional exploration using the five senses (e.g., experiencing an apple ‘for the first time’)
5	Self-knowledge Self-management Social consciousness Interpersonal relationship Responsible decision-making Attentional focus	‘Discover Intergalactic Objects’ ‘The Party in Space’ ‘My Superpowers’: Imagining new superpowers, naming creatively	Attention games like ‘find lost objects’ in the ‘galaxy of feelings and behaviors’ Pair exercise to create a party theme (e.g., dance, game, or theater) and culminate in a large group ‘party’
6	Self-knowledge Self-management Social consciousness Interpersonal relationship Responsible decision-making Attentional focus	‘The Intergalactic School’ ‘A House on Mars’ ‘My Superpowers’ (Super Ideas and Super Happiness)	Reflection on school and friendships through role-play Group creation of ‘ideal school and home’ via role-plays, drawings, dramatizations, and creative writing
7	Self-knowledge Self-management Social consciousness Interpersonal relationship Responsible decision-making	‘A Statue in the Intergalactic Galaxy’ ‘A Toast to the Intergalactic Union’ ‘My Superpowers’ (Super Strength and Super Protection)	Balance games in pairs (e.g., statue, ‘no smiling’ challenge) Imagery exercise imagining ‘your own planet’ with detailed sensory descriptions Collaborative addition of each ‘planet’ to a ‘galaxy of feelings and behaviors’ with distinct roles
8	Self-knowledge Self-management Social consciousness Interpersonal relationship	‘Intergalactic Friendship’ ‘Emotions in Space’ ‘My Superpowers’: Create and name new superpowers ‘Intergalactic Program Diploma’ ceremony	Draw and reflect on an ‘intergalactic friend’ with pair and group sharing on associated characteristics Emotional expression and movement through role-play and freeze exercises

### 2.3 Measure

The BRIEF (Behavior Rating Inventory of Executive Function) is a questionnaire designed for parents and teachers to evaluate EFs related behaviors in both home and school environments (Gioia et al., 2000). Each version of the questionnaire consists of 86 items divided into eight clinical subscales that encompass essential aspects of EFs: inhibition, shifting, emotional control, initiation, working memory, planning/organization, organization of materials, and monitoring. The obtained scores facilitate the calculation of an overall score known as the Global Executive Composite (GEC).

For the current analysis, only raw scores on the GEC were computed and analyzed. According to the BRIEF manual, higher GEC scores indicate greater difficulties in executive functioning, whereas lower scores reflect fewer observed difficulties. It is also important to note that the BRIEF does not assess the developmental progression of EFs across ages but identifies difficulties or impairments in EFs relative to developmental expectations. The instrument is sensitive to clinically relevant changes over time (e.g., following intervention or in response to contextual changes), but it is not designed to measure age-related cognitive maturation per se. Internal consistency ranged from 0.80 to 0.98 (Cronbach’s *α*), and test–retest reliability ranged from 0.76 to 0.88. A European Portuguese translation was used for this study (Barbosa et al., 2011). The primary focus was on the overall executive functioning scale, specifically, the GEC scores derived from parent and teacher reports.

### 2.4 Blinding

Participants, teachers, and parents were blinded to the study’s hypothesis. However, the participants and teachers in the intervention group were aware of the general content of the sessions.

### 2.5 Procedure

The intervention program was conducted in a group format (by class) over eight weekly sessions during the second and third terms of the academic year. Each session lasted 60 min and was facilitated within the school setting by two trained psychologists.

Both intervention and control groups were assessed at two time points: pre-intervention (Time 1, T1) and post-intervention (Time 2, T2). The T1 assessments were conducted between January and February 2024, while the post-intervention assessments (T2) took place in May 2024. Caregivers and teachers were asked to complete the BRIEF (i.e., the standardized questionnaire for assessing EFs) at T1 and T2, providing a comprehensive evaluation of potential changes in EFs across time. Treatment fidelity was maintained at 100% across all groups. When the session could not be completed within the allocated time, the remaining content was addressed in the following session.

Participants were recruited in accordance with the ethical standards of the European Union Agency for Fundamental Rights and the Declaration of Helsinki (World Medical Association). The Ethics Committee granted ethical approval for the study at the first author’s institution. Informed consent was obtained from the legal guardians of participating children, and child assent was also ensured.

## 3 Results

A 2 (Group: Intervention vs. Control) × 2 (Time: Pre-intervention vs. Post-intervention) repeated measures ANCOVA was conducted to examine changes in EFs over time, based on ratings from teachers and caregivers while controlling for participants’ age as a covariate (cf. Tables 3, 4).

**Table 3:** Descriptive statistics for executive function scores by group (Intervention vs. Control) – teacher and caregiver ratings.

Group	Time point	Measure	*M*	*SD*
Intervention group	Pre-intervention	Teacher rating	110.92	22.70
		Caregiver rating	137.93	23.22
	Post-intervention	Teacher rating	109.98	24.61
		Caregiver rating	123.60	22.72
Waiting list control group	Pre-intervention	Teacher rating	100.28	16.04
		Caregiver rating	112.79	21.72
	Post-intervention	Teacher rating	97.23	13.11
		Caregiver rating	117.03	16.81

**Table 4:** Repeated-measures ANCOVA results for executive function scores rated by teachers and caregivers in a 2 × 2 mixed design (Time × Group), with age as a covariate.

	df	*F*	*p*	η^2^ₚ
Teacher ratings	1, 90			
Time	1, 90	0.25	0.62	0.003
Time × Group	1, 90	0.02	0.90	<0.001
Age (covariate)	1, 90	0.71	0.40	0.008
Time × Age	1, 90	0.45	0.50	0.005
Caregiver ratings				
Time	1, 91	0.37	0.54	0.004
Time × Group	1, 91	11.00	0.001	0.108
Age (covariate)	1, 91	1.05	0.31	0.011
Time × Age	1, 91	0.81	0.37	0.009

η^2^ₚ, partial eta squared. Group refers to intervention vs. control condition. Time refers to the two measurement points: pre-intervention and post-intervention. Ratings are based on BRIEF – Global Executive Composite scores.

For the teacher ratings, the ANCOVA results indicated a non-significant effect of time *F*(1, 90) = 0.245, *p* = 0.622, η^2^ₚ = 0.003, suggesting no statistically significant overall change in EFs scores from pre-intervention to post-intervention across both groups. The main effect of age was not significant, *F*(1, 90) = 0.706, *p* = 0.403, η^2^ₚ = 0.008, and the time × age interaction was also not significant, *F*(1, 90) = 0.450, *p* = 0.504, η^2^ₚ = 0.005. The interaction between time and group was not significant either, *F*(1, 90) = 0.015, *p* = 0.904, η^2^ₚ < 0.001, indicating that the pattern of change in EFs scores over time was similar for both the intervention and control groups. Therefore, according to teacher ratings, there is no evidence that the intervention produced a differential effect compared to the control group.

For caregivers, the analysis revealed no significant main effect of time, *F*(1, 91) = 0.374, *p* = 0.542, η^2^ₚ = 0.004, suggesting that, overall, EFs scores did not significantly change from pre- to post-intervention across groups after controlling for age. However, a significant time × group interaction was found, *F*(1, 91) = 10.996, *p* = 0.001, η^2^ₚ = 0.108, which indicates that the two groups followed significantly different trajectories over time. Specifically, only the intervention group showed a reduction in EFs scores from pre- to post-intervention, suggesting improved executive functioning, whereas the control group remained relatively stable. The main effect of age was not significant, *F*(1, 91) = 1.046, *p* = 0.309, η^2^ₚ = 0.011, and the interaction between time and age was also non-significant, *F*(1, 91) = 0.807, *p* = 0.371, η^2^ₚ = 0.009, indicating that age was neither associated with EFs scores overall nor did it influence changes in EFs scores over time.

## 4 Discussion

Although evidence-based SEL programs have shown potential for promoting social–emotional and cognitive skills, critical gaps remain in the literature, particularly regarding their impact on EFs. While mindfulness-based interventions have demonstrated positive outcomes on EFs, the classification of these interventions as a subset of SEL programs is debated. Furthermore, the generalization of these results is often limited by contextual and cultural differences, highlighting the need for studies to explore their adaptability and effectiveness in diverse contexts and populations. This pilot study examined the effects of an SEL program, *The Intergalactic World*, on the development of EFs in elementary school children. Using a cluster-randomized controlled trial design, 96 students from 1st to 4th grade were assigned to intervention and control groups. The SEL program included eight sessions promoting self-regulation and emotional control skills. The results revealed a significant interaction effect between time and group for caregiver ratings of EFs, suggesting that children in the intervention group showed greater progress in EFs compared to those in the control group. However, these significant effects were not observed in teacher ratings, which did not align with the anticipated results.

Caregivers’ ratings of EFs in the present study align with previous studies demonstrating the potential of SEL programs to enhance children’s cognitive and emotional development (Durlak et al., 2011; Taylor et al., 2017). Thus, compared to the control group, the unique trajectory observed in the intervention group underscores the idea that structured SEL programs can effectively support socio-emotional development and transfer to cognitive skills such as EFs. These findings emphasize the potential for integrating SEL programs into school curricula to address broader developmental needs beyond academic performance.

From a sociocultural perspective, particularly Vygotsky’s theoretical framework (Vygotsky, 1962), the observed improvements in EFs among children in the intervention group can be interpreted as the result of mediated learning processes within structured social contexts. Vygotsky emphasized that higher-order cognitive functions, such as self-regulation, emerge first through social interaction and are then internalized. SEL programs like *The Intergalactic World*, which employ guided activities, function as cultural tools that scaffold the development of these regulatory capacities. Through repeated, supported engagement in these practices, children may gradually internalize executive processes. This may also explain why caregivers, who observe children across varied and dynamic environments, reported significant gains in EFs.

In addition, research emphasizes the reciprocal relationship between EFs and social–emotional skills. Functions such as self-regulation, working memory, and cognitive flexibility are essential for developing social–emotional skills, with children demonstrating stronger EFs skills typically exhibiting better emotional and behavioral regulation. SEL programs incorporating EFs development have been shown to improve social–emotional and cognitive skills by helping children process social information, make decisions, and resolve conflicts more effectively. This integrated approach highlights the importance of addressing both EFs and social–emotional simultaneously, reinforcing the need for educational strategies that promote the development of both domains (O'Conner et al., 2017).

The lack of significant changes in EFs scores from teacher evaluations highlights an important discrepancy. This difference likely stems from contextual factors: caregivers observe children in varied and less structured environments, allowing them to notice subtle changes in emotional and self-regulation behaviors (Toplak et al., 2013). In contrast, teachers operate in performance-focused classroom settings, where the predominant focus on academic performance and structured interactions may mask such changes (Cristóvão et al., 2017; Weissberg et al., 2015). Furthermore, although short-term SEL interventions may lead to initial improvements, longer programs or those that target broader competencies such as problem-solving and communication are more likely to generate stronger and longer-lasting effects in classroom settings (Aber et al., 2003; Linares et al., 2005).

Although the findings suggest that participation in *The Intergalactic World* program significantly enhanced EFs among Portuguese first- to fourth-grade students, with improvements reflected in parent ratings for the intervention group compared to the control group, several limitations of the study warrant consideration. First, the short duration of the intervention (eight sessions) could constrain the program’s impact. Research suggests that longer and more comprehensive programs produce more substantial and sustained changes, particularly within structured environments such as classrooms (Aber et al., 2003; Linares et al., 2005).

Second, although the study included ecologically valid measures of EFs, such as teacher and caregiver ratings, integrating performance-based assessments, qualitative approaches (e.g., focus groups), and self-report questionnaires for children could provide more nuanced insights into the program’s effects. Self-report measures would allow children to express their perceptions of their own EFs, offering an additional insight that complements external evaluations by caregivers and teachers.

A further limitation concerns the group allocation procedure. Although a cluster-randomized design was employed, the randomization process was constrained by logistical requirements imposed by the school. As a result, the intervention group included a larger number of participants than the control group, and the age distribution between groups was not fully balanced. These factors may have introduced confounding variables that limit the internal validity of group comparisons.

Additionally, the study was conducted in a single private school, which may restrict the generalizability of the findings. Students in private educational settings often differ from those in public schools in terms of socioeconomic background, access to resources, and educational environment. These contextual factors may influence both baseline functioning and responsiveness to intervention. As such, caution is warranted when applying these results to more diverse or representative populations. Future research should aim to replicate and extend these findings across multiple school types and demographic contexts to enhance external validity.

Suggestions for future studies include using a randomized study design with a larger and more diverse sample. These studies should also consider evaluating the long-term effectiveness of the intervention through follow-ups conducted 1–2 years after the program’s completion. Additionally, future research should prioritize the rigorous assessment and monitoring of program implementation and examine contextual and environmental factors that may influence program outcomes (Durlak et al., 2011). Future programs could also include specific strategies to train teachers in identifying and monitoring subtle changes in executive and socio-emotional behaviors, which may enhance their ability to perceive the program benefits.

From a practical perspective, the findings suggest that SEL programs can significantly enhance EFs development when delivered in contexts that allow for flexibility and varied interactions. For teachers, embedding SEL strategies into daily classroom practices and providing specific training in recognizing socio-emotional and cognitive changes could enhance the effectiveness of such programs in structured school settings. Additionally, fostering collaborative feedback mechanisms between teachers and caregivers may help bridge the perception gap and provide a holistic understanding of children’s development.

In summary, this study contributes valuable evidence supporting the efficacy of SEL programs like *The Intergalactic World* in enhancing EFs in elementary school children, particularly as reported by caregivers. The findings underscore the importance of considering contextual and observational factors in evaluating SEL outcomes and highlight the need for longer and more integrative interventions. Addressing these limitations and employing diverse methodologies will provide a stronger foundation for integrating SEL into education systems and maximizing its benefits for children’s cognitive and socio-emotional development.

Although preliminary, these findings underscore the potential value of integrating structured SEL programs into early primary education to support executive function development. The effectiveness of such programs may be further enhanced through coordinated efforts between educators and caregivers. At the policy level, these results suggest the relevance of embedding SEL within national curricular frameworks, investing in ongoing professional development for teachers focused on socio-emotional and cognitive development, and implementing scalable systems for monitoring behavioral and regulatory outcomes over time.

## Data Availability

The raw data supporting the conclusions of this article will be made available by the authors, without undue reservation.

## References

[ref1] Aber J. L. Brown J. L. Jones S. M. (2003). Developmental trajectories toward violence in middle childhood: course, demographic differences, and response to school-based intervention. Dev. Psychol. 39:324. doi: 10.1037//0012-1649.39.2.324 12661889

[ref2] Anderson V. Levin H. S. Jacobs R. (2002). “Executive functions after frontal lobe injury: A developmental perspective” in Principles of frontal lobe function. eds. Stuss D. T. Knight R. T. (Oxford University), 504–527.

[ref3] Antunes R. Alexandre J. Guedes M. Filipe M. G. Veríssimo M. (2023). Assessing the benefits of the “intergalactic world” social emotional learning program for 8–12-year-old children in Portugal: perspectives from teachers and caregivers. Front. Psychol. 14:1233335. doi: 10.3389/fpsyg.2023.1233335 37701869 PMC10493273

[ref4] Antunes R. Guedes M. Alexandre J. Veríssimo M. (2022). Benefícios de um novo programa de aprendizagem socioemocional na redução da sintomatologia psicopatológica e na promoção das competências socioemocionais globais, na perspetiva das crianças. Psicologia 36, 119–135. doi: 10.17575/psicologia.1813

[ref5] Barbosa A. Teles S. Vicente S. G. (2011). Behavior Rating of Executive Function (BRIEF): European Portuguese - Parental Version [working research version constructed based on the BRIEF of Gioia, Isquith, Guy, and Kenworthy, 2000]. Center for Psychology, Faculty of Psychology and Educational Sciences, University of Porto, Porto, Portugal.

[ref6] Black D. S. Fernando R. (2014). Mindfulness training and classroom behavior among lower-income and ethnic minority elementary school children. J. Child Fam. Stud. 23, 1242–1246. doi: 10.1007/s10826-013-9784-4 25624749 PMC4304073

[ref7] Cipriano C. Strambler M. J. Naples L. H. Ha C. Kirk M. Wood M.. (2023). The state of evidence for social and emotional learning: a contemporary meta-analysis of universal school-based SEL interventions. Child Dev. 94, 1181–1204. doi: 10.1111/cdev.13968 37448158

[ref8] Clark C. Prior M. Kinsella G. (2002). The relationship between executive function abilities, adaptive behaviour, and academic achievement in children with externalising behaviour problems. J. Child Psychol. Psychiatry Allied Discip. 43, 785–796. doi: 10.1111/1469-7610.00084 12236613

[ref9] Collaborative for Academic, Social, and Emotional Learning (2012). 2013 CASEL *guide: Effective social and emotional learning programs—Preschool and elementary*. School Edn. Chicago, IL: Author.

[ref10] Cristóvão A. M. Candeias A. A. Verdasca J. (2017). Social and emotional learning and academic achievement in Portuguese schools: a bibliometric study. Front. Psychol. 8:1913. doi: 10.3389/fpsyg.2017.01913 29167650 PMC5682338

[ref11] Crooks C. V. Bax K. Delaney A. Kim H. Shokoohi M. (2020). Impact of MindUP among young children: improvements in behavioral problems, adaptive skills, and executive functioning. Mindfulness 11, 2433–2444. doi: 10.1007/s12671-020-01460-0

[ref12] Diamond A. Lee K. (2011). Interventions shown to aid executive function development in children 4 to 12 years old. Science 333, 959–964. doi: 10.1126/science.1204529 21852486 PMC3159917

[ref13] Dias N. M. Trevisan B. T. León C. B. R. Prust A. P. Seabra A. G. (2017). Can executive functions predict behavior in preschool children? Psychol. Neurosci. 10:383. doi: 10.1037/pne0000104

[ref14] Domitrovich C. E. Durlak J. A. Staley K. C. Weissberg R. P. (2017). Social-emotional competence: an essential factor for promoting positive adjustment and reducing risk in school children. Child Dev. 88, 408–416. doi: 10.1111/cdev.12739 28213889

[ref15] Durlak J. A. Mahoney J. L. Boyle A. E. (2022). What we know, and what we need to find out about universal, school-based social and emotional learning programs for children and adolescents: a review of meta-analyses and directions for future research. Psychol. Bull. 148:765. doi: 10.1037/bul0000383

[ref16] Durlak J. A. Weissberg R. P. Dymnicki A. B. Taylor R. D. Schellinger K. B. (2011). The impact of enhancing students’ social and emotional learning: a meta-analysis of school-based universal interventions. Child Dev. 82, 405–432. doi: 10.1111/j.14678624.2010.01564.x 21291449

[ref17] Fernández-Martín F. D. Romero-Rodríguez J. M. Marín-Marín J. A. Gómez-García G. (2021). Social and emotional learning in the Ibero-American context: a systematic review. Front. Psychol. 12:738501. doi: 10.3389/fpsyg.2021.738501 34659053 PMC8516388

[ref18] Ferraioli S. J. Harris S. L. (2013). Comparative effects of mindfulness and skills-based parent training programs for parents of children with autism: feasibility and preliminary outcome data. Mindfulness 4, 89–101. doi: 10.1007/s12671-012-0099-0

[ref19] Flook L. Smalley S. L. Kitil M. J. Galla B. M. Kaiser-Greenland S. Locke J.. (2010). Effects of mindful awareness practices on executive functions in elementary school children. J. Appl. Sch. Psychol. 26, 70–95. doi: 10.1080/15377900903379125

[ref20] Gioia G. A. Guy S. C. Isquith P. K. Kenworthy L. (2000). Behavior rating inventory of executive function. Florida, FL: PAR.

[ref21] Grazzani I. Agliati A. Cavioni V. Conte E. Gandellini S. Lupica Spagnolo M.. (2022). Adolescents' resilience during COVID-19 pandemic and its mediating role in the association between SEL skills and mental health. Front. Psychol. 13:801761. doi: 10.3389/fpsyg.2022.801761 35197901 PMC8860228

[ref22] Hassani S. (2024). Fostering social-emotional competencies to improve social functioning, social inclusion, and school well-being: results of a cluster non-randomized pilot study. Ment. Health Prev. 36:200365. doi: 10.1016/j.mhp.2024.200365

[ref23] Huguet A. Ruiz D. M. Haro J. M. Alda J. A. (2017). A pilot study of the efficacy of a mindfulness program for children newly diagnosed with attention-deficit hyperactivity disorder: impact on core symptoms and executive functions. Int. J. Psychol. Psychol. Ther. 17, 305–316.

[ref24] Jurado M. B. Rosselli M. (2007). The elusive nature of executive functions: a review of our current understanding. Neuropsychol. Rev. 17, 213–233. doi: 10.1007/s11065-007-9040-z 17786559

[ref25] Kankaraš M. Suarez-Alvarez J. (2019). Assessment framework of the OECD study on social and emotional skills. OECD Educ. Work. Papers 207, 1–109. doi: 10.1787/5007adef-en

[ref26] Klingbeil D. A. Renshaw T. L. Willenbrink J. B. Copek R. A. Chan K. T. Haddock A.. (2017). Mindfulness based interventions with youth: a comprehensive meta-analysis of group-design studies. J. Sch. Psychol. 63, 77–103. doi: 10.1016/j.jsp.2017.03.006 28633940

[ref27] Lawlor M. S. (2016). “Mindfulness and social emotional learning (SEL): a conceptual framework” in Handbook of mindfulness in education. eds. Schonert-Reichl K. A. Roeser R. W. (New York: Springer), 65–80.

[ref28] Lewis C. Koyasu M. Oh S. Ogawa A. Short B. Huang Z. (2009). Culture, executive function, and social understanding. New Dir. Child Adolesc. Dev. 2009, 69–85. doi: 10.1002/cd.236 19306275

[ref29] Linares L. O. Rosbruch N. Stern M. B. Edwards M. E. Walker G. Abikoff H. B.. (2005). Developing cognitive-social-emotional competencies to enhance academic learning. Psychol. Sch. 42, 405–417. doi: 10.1002/pits.20066

[ref30] Mahoney J. L. Weissberg R. P. Greenberg M. T. Dusenbury L. Jagers R. J. Niemi K.. (2021). Systemic social and emotional learning: promoting educational success for all preschool to high school students. Am. Psychol. 76, 1128–1142. doi: 10.1037/amp0000701 33030926

[ref31] Miyake A. Friedman N. P. Emerson M. J. Witzki A. H. Howerter A. Wager T. D. (2000). The unity and diversity of executive functions and their contributions to complex “frontal lobe” tasks: a latent variable analysis. Cogn. Psychol. 41, 49–100. doi: 10.1006/ cogp.1999.0734 10945922 10.1006/cogp.1999.0734

[ref32] Moreira P. Crusellas L. Sá I. Gomes P. Matias C. (2010). Evaluation of a manual-based programme for the promotion of social and emotional skills in elementary school children: results from a 4-year study in Portugal. Health Promot. Int. 25, 309–317. doi: 10.1093/heapro/daq029 20418389

[ref33] O'Conner R. De Feyter J. Carr A. Luo J. L. Romm H. (2017). A review of the literature on social and emotional learning for students ages 3-8: Characteristics of effective social and emotional learning programs (Part 1 of 4). REL 2017-245. REL Mid-Atlantic.Washington, DC:Regional Educational Laboratory Mid-Atlantic.

[ref34] OECD (2024). Social and emotional skills for better lives: Findings from the OECD survey on social and emotional skills 2023. Paris: OECD Publishing.

[ref35] Office of the Surgeon General (2021). Protecting youth mental health: The U.S. surgeon general’s advisory. Washington (DC): US Department of Health and Human Services. 34982518

[ref36] Raimundo R. Oliveira S. Roberto M. S. Marques-Pinto A. (2024). Effects of a social–emotional learning intervention on social–emotional competencies and behavioral problems in elementary students amid COVID-19. Int. J. Environ. Res. Public Health 21:1223. doi: 10.3390/ijerph21091223 39338106 PMC11432258

[ref37] Raveepatarakul J. Suttiwan P. Iamsupasit S. Mikulas W. L. (2014). A mindfulness enhancement program for 8 to 11 year-old Thai children: effects on mindfulness and depression. J. Health Res. 28, 335–341.

[ref38] Riggs N. R. Blair C. B. Greenberg M. T. (2004). Concurrent and 2-year longitudinal relations between executive function and the behavior of 1st and 2nd grade children. Child Neuropsychol. 9, 267–276. doi: 10.1076/chin.9.4.267.23513 14972705

[ref39] Riggs N. R. Jahromi L. B. Razza R. P. Dillworth-Bart J. E. Mueller U. (2006). Executive function and the promotion of social–emotional competence. J. Appl. Dev. Psychol. 27, 300–309. doi: 10.1016/j.appdev.2006.04.002

[ref40] Ritter A. Alvarez I. (2020). Mindfulness and executive functions: making the case for elementary school practice. Eur. J. Investig. Health Psychol. Educ. 10, 544–553. doi: 10.3390/ejihpe10010039 PMC8314250 34542502

[ref41] Sanders M. R. (2008). Triple P-positive parenting program as a public health approach to strengthening parenting. J. Fam. Psychol. 22:506. doi: 10.1037/0893-3200.22.3.506 18729665

[ref42] Semple R. J. Droutman V. Reid B. A. (2017). Mindfulness goes to school: things learned (so far) from research and real-world experiences. Psychol. Sch. 54, 29–52. doi: 10.1002/pits.21981 28458403 PMC5405439

[ref43] Silver C. H. (2014). Sources of data about children’s executive functioning: review and commentary. Child Neuropsychol. 20, 1–13. doi: 10.1080/09297049.2012.727793 23030631

[ref44] Singh N. Duraiappah A. K. (2020). Rethinking learning: A review of social and emotional learning frameworks for education systems. New Delhi: UNESCO MGIEP.

[ref45] Smith T. Panfil K. Bailey C. Kirkpatrick K. (2019). Cognitive and behavioral training interventions to promote self-control. J. Exp. Psychol. Anim. Learn. Cogn. 45, 259–279. doi: 10.1037/xan0000208 31070430 PMC6716382

[ref46] Soriano-Ferrer M. Félix-Mateo V. Begeny J. C. (2014). Executive function domains among children with ADHD: do they differ between parents and teachers ratings? Procedia. Soc. Behav. Sci. 132, 80–86. doi: 10.1016/j.sbspro.2014.04.281

[ref47] Sudikoff E. L. Bertolin M. Lordo D. N. Kaufman D. A. S. (2015). Relationships between executive function and emotional regulation in healthy children. J. Neurol. Psychol 2, 1–8.

[ref48] Tan A. Delgaty L. Steward K. Bruner M. (2018). Performance-based measures and behavioral ratings of executive function in diagnosing attention-deficit/hyperactivity disorder in children. Atten. Defic. Hyperact. Disord. 10, 309–316. doi: 10.1007/s12402-018-0256-y 29663184

[ref49] Taylor R. D. Oberle E. Durlak J. A. Weissberg R. P. (2017). Promoting positive youth development through school-based social and emotional learning interventions: a meta-analysis of follow-up effects. Child Dev. 88, 1156–1171. doi: 10.1111/cdev.12864 28685826

[ref50] Thierry K. L. Bryant H. L. Nobles S. S. Norris K. S. (2016). Two year impact of a mindfulness-based program on preschoolers’ self regulation and academic performance. Early Educ. Dev. 27, 805–821. doi: 10.1080/10409289.2016.1141616

[ref51] Thompson W. K. Barch D. M. Bjork J. M. Gonzalez R. Nagel B. J. Nixon S. J.. (2019). The structure of cognition in 9 and 10 year-old children and associations with problem behaviors: findings from the ABCD study’s baseline neurocognitive battery. Dev. Cogn. Neurosci. 36:100606. doi: 10.1016/j.dcn.2018.12.004 30595399 PMC6676481

[ref52] Toplak M. E. West R. F. Stanovich K. E. (2013). Practitioner review: do performance-based measures and ratings of executive function assess the same construct? J. Child Psychol. Psychiatry 54, 131–143. doi: 10.1111/jcpp.12001 23057693

[ref53] Vickery C. E. Dorjee D. (2016). Mindfulness training in primary schools decreases negative affect and increases meta-cognition in children. Front. Psychol. 6:2025. doi: 10.3389/fpsyg.2015.02025 26793145 PMC4709470

[ref54] Vygotsky L. (1962). Thought and language. Boston Review 29. doi: 10.1037/11193-000

[ref55] Webster-Stratton C. (2016). “The Incredible Years® series: a developmental approach,” in *Family-Based Prevention Programs for children and Adolescents: Theory, Research, and Large-Scale Dissemination*, ed. Ryzin M. J. Van Kumpfer K. L. Fosco G. M. Greenberg M. T. (New York: Psychology Press), 42–67.

[ref56] Weissberg R. P. (2019). Promoting the social and emotional learning of millions of school children. Perspect. Psychol. Sci. 14, 65–69. doi: 10.1177/1745691618817756 30799753

[ref57] Weissberg R. P. Durlak J. A. Domitrovich C. E. Gullotta T. P. (2015). “Social and emotional learning: Past, present, and future” in Handbook of social and emotional learning: Research and practice. eds. Durlak J. A. Domitrovich C. E. Weissberg R. P. Gullotta T. P. (New York: The Guildford Press), 3–19.

[ref58] Zelazo P. D. Lyons K. E. (2012). The potential benefits of mindfulness training in early childhood: a developmental social cognitive neuroscience perspective. Child Dev. Perspect. 6, 154–160. doi: 10.1111/j.1750-8606.2012.00241.x

[ref59] Zhang L. Ma Y. (2023). A study of the impact of project-based learning on student learning effects: a meta-analysis study. Front. Psychol. 14:1202728. doi: 10.3389/fpsyg.2023.1202728 37564309 PMC10411581

